# Association Between Telomere Length and Skin Cancer and Aging: A Mendelian Randomization Analysis

**DOI:** 10.3389/fgene.2022.931785

**Published:** 2022-07-12

**Authors:** Nannan Song, Yankun Cui, Wang Xi

**Affiliations:** Jiangxi University of Chinese Medicine, Nanchang, China

**Keywords:** telomere length, skin cancer, skin aging, mendelian randomization, age

## Abstract

**Background:** Telomere shortening is a hallmark of cellular senescence. However, telomere length (TL)-related cellular senescence has varying effects in different cancers, resulting in a paradoxical relationship between senescence and cancer. Therefore, we used observational epidemiological studies to investigate the association between TL and skin cancer and aging, and to explore whether such a paradoxical relationship exists in skin tissue.

**Methods:** This study employed two-sample Mendelian randomization (MR) to analyze the causal relationship between TL and skin cancer [melanoma and non-melanoma skin cancers (NMSCs)] and aging. We studied single nucleotide polymorphisms (SNPs) obtained from pooled data belonging to genome-wide association studies (GWAS) in the literature and biobanks. Quality control was performed using pleiotropy, heterogeneity, and sensitivity analyses.

**Results:** We used five algorithms to analyze the causal relationship between TL and skin aging, melanoma, and NMSCs, and obtained consistent results. TL shortening reduced NMSC and melanoma susceptibility risk with specific odds ratios (ORs) of 1.0344 [95% confidence interval (CI): 1.0168–1.0524, *p* = 0.01] and 1.0127 (95% CI: 1.0046–1.0209, *p* = 6.36E-07), respectively. Conversely, TL shortening was validated to increase the odds of skin aging (OR = 0.96, 95% CI: 0.9332–0.9956, *p* = 0.03). Moreover, the MR-Egger, maximum likelihood, and inverse variance weighted (IVW) methods found significant heterogeneity among instrumental variable (IV) estimates (identified as MR-Egger skin aging Q = 76.72, *p* = 1.36E-04; melanoma Q = 97.10, *p* = 1.62E-07; NMSCsQ = 82.02, *p* = 1.90E-05). The leave-one-out analysis also showed that the SNP sensitivity was robust to each result.

**Conclusion:** This study found that TL shortening may promote skin aging development and reduce the risk of cutaneous melanoma and NMSCs. The results provide a reference for future research on the causal relationship between skin aging and cancer in clinical practice.

## 1 Introduction

Telomeres are DNA–protein complexes, located at the chromosome ends of eukaryotic cells, that protect the chromosomes from degradation and fusion (Shay, 2018). Defective telomere function has been shown to lead to genetic instabilities in cancer, with the telomeres shortening as cells age (Blasco, 2005). Telomere length (TL) in cells has been extensively studied as an aging biomarker and risk factor for age-related diseases. However, the extent to which TL can reflect cancer relevance remains unclear (Arsenis et al., 2017). Telomere shortening accelerates skin aging while acting as a mitotic clock, preventing abnormal proliferation in cancer (Buckingham and Klingelhutz, 2011). Skin is a highly self-renewing tissue that must undergo extensive proliferation throughout an organism’s life cycle. It is generally believed that aging caused by telomere shortening can increase cancer risk. However, many studies have found that cancers such as melanoma may occur due to excessive telomere lengthening (Ismail et al., 2021). Clarifying this contradiction requires further clinical and epidemiological research.

The skin is the largest organ in the human body, accounting for approximately 15% of an adult’s body weight. Skin aging is a major problem and involves multiple complex factors, such as cellular DNA damage and changes in mitochondrial function (Lowry, 2020). As one of the most common cancers, the prevalence of skin cancer has been increasing over the past 3 decades. Skin cancers are mainly divided into melanoma and non-melanoma skin cancers (NMSCs); the latter includes basal cell carcinoma and squamous cell carcinoma cancer (Linares et al., 2015). According to the World Health Organization, as many as 60,000 people worldwide die of skin cancer each year, with melanoma being responsible for most cancer-related deaths. Therefore, exploring the correlation between multiple risk factors, skin cancer, and aging is necessary.

**FIGURE 1:**
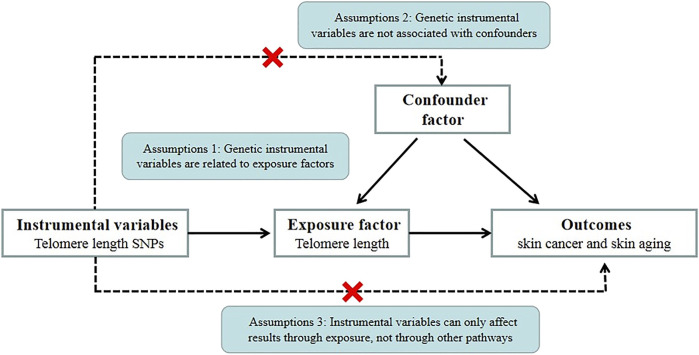
MR basic requirements framework. Two-sample MR studies need to satisfy three assumptions. Assumption 1: Instrumental variables should be associated with telomere length. Assumption 2: The selected instrumental variables should be independent of confounders. Assumption 3: Instrumental variables should affect outcomes only through exposure and not through direct correlation.

We conducted Mendelian randomization (MR) analysis to explore the causal relationship between TL and skin aging and the risk of skin cancer (NMSCs and melanoma). MR is also known as “Mendelian deconfounding” because it aims to give an estimate of causality without bias due to confounders. The instrumental variables (IVs) in MR studies must satisfy three core assumptions: 1) genetic IVs are related to exposure factors, 2) genetic IV formation can be regarded as a random assignment and has nothing to do with confounding factors, and 3) IVs can only be associated with exposure factors that affect the outcomes (Bowden and Holmes, 2019) (Figure 1). To avoid violating the assumptions of MR, first, we need to perform horizontal pleiotropy analysis to prevent IVs from directly affecting the results without exposure factor (Morrison et al., 2020). Second, removing SNPs in linkage disequilibrium (LD) (Cheng et al., 2020), avoiding IVs associated with causal variants may contribute to confounders. Third, intrinsic differences between populations (confounding factors) can be mitigated by restricting study populations to the same ethnic background.

## 2 Materials and Methods

### 2.1 Study Design Overview

**FIGURE 2:**
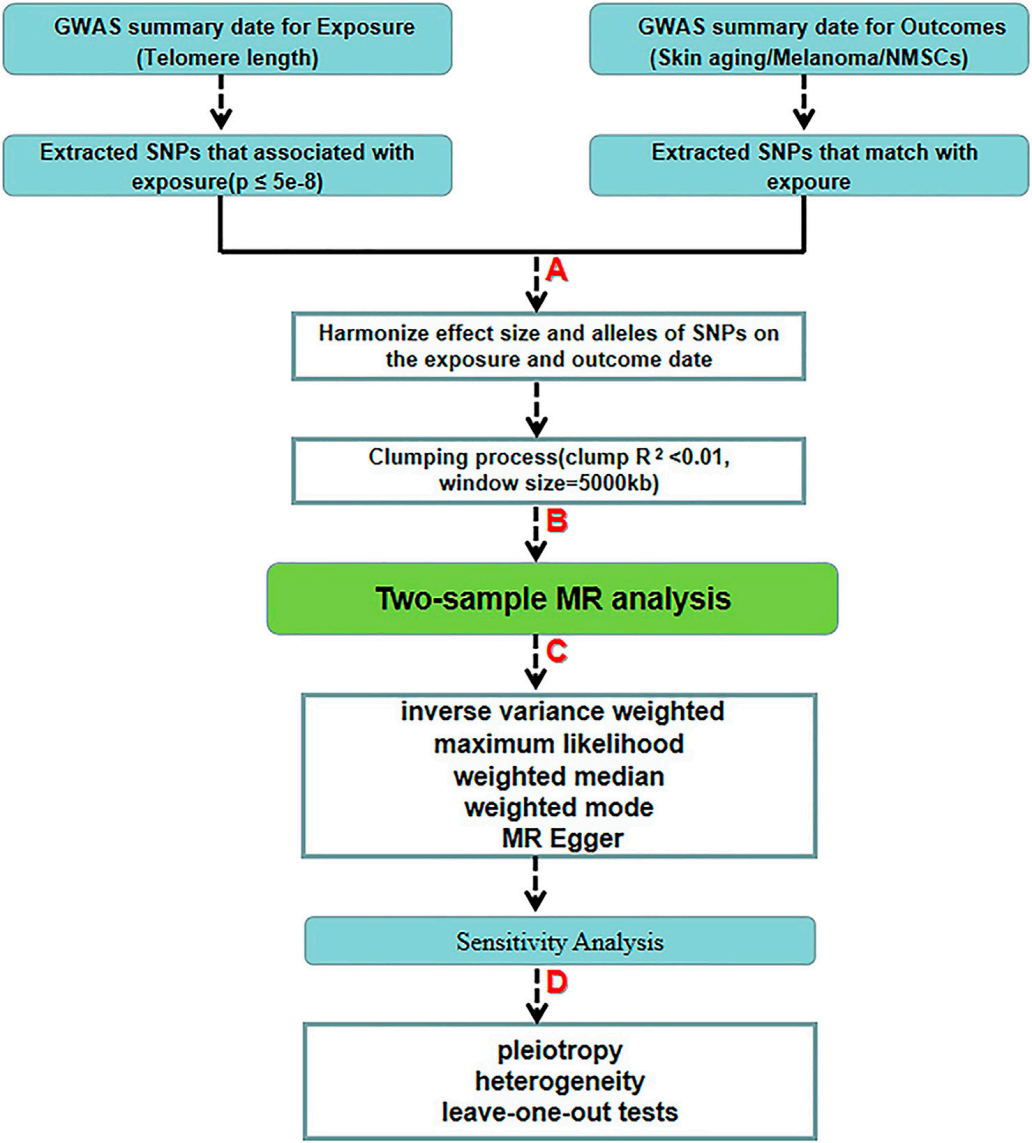
Schematic diagram of the two-sample-MR analysis process. **(A)** Screening from GWAS data for selected SNPs significantly associated with exposure (*p* < 5.00 E-08 at genome-wide threshold). **(B)** Obtaining independent SNPs and examining effect size outliers with linkage disequilibrium (LD) with r < 0.01 or physical distance greater than 5000 kb. **(C)** Two-sample MR analysis by five algorithms. **(D)** Quality control by sensitivity analysis.

An overview of the study design is drawn in Figure 2. we used a two-sample MR study to explore possible causal relationships between our study’s exposure and outcome. We used genome-wide association study (GWAS) datasets to estimate the effect of the exposure (TL) on the outcomes (skin cancer and skin aging). We selected single nucleotide polymorphisms (SNPs) closely associated with TL as IVs based on previously published GWAS databases and literature reports. The effects of the IVs on the exposure and outcomes were obtained from two independent samples. Ethical approval was provided in the original article for the GWAS-pooled dataset used in this study. Therefore, informed consent was no longer required.

### 2.2 Genetic Instrument Selection

Based on literature reports, SNP sites related to TL were screened. The SNPs associated with TL used in the present study came from a large GWAS of 7859 individuals from Europe. TL was measured in a mixed population of leukocytes using the established quantitative polymerase chain reaction technique, which expresses TL as the ratio of the number of telomere repeats to single-copy genes. Normalizing leukocyte TL measurements required the use of calibration samples or the quantification of a standard curve (Li et al., 2020). Statistics using cohort data identified 17 genome-wide significant loci, including several novel genes (*SENP7*, *MOB1B*, *CARMIL1*, *PRRC2A*, *TERF2*, and *RFWD3*), and confirmed the presence of other relevant genes (Table 1).

**TABLE 1:** Association of 42 TL SNPs.

SNP ID	Closest gene	EA	NEA	EAF	Beta	SE	*p* value
rs10936600	LRRC34	T	A	0.2430	−0.0858	0.0057	6.42E-51
rs7705526	TERT	A	C	0.3283	0.0820	0.0058	4.82E-45
rs4691895	TERT	C	G	0.7829	0.0577	0.0061	1.47E-21
rs9419958	STN1	C	T	0.8616	−0.0636	0.0071	4.77E-19
rs75691080	STMN3	T	C	0.0912	−0.0671	0.0089	5.75E-14
rs59294613	POT1	A	C	0.2928	−0.0407	0.0055	1.12E-13
rs8105767	ZNF208	G	A	0.2887	0.0392	0.0054	5.21E-13
rs3219104	PARP1	C	A	0.8302	0.0417	0.0064	9.31E-11
rs2736176	PRRC2A	C	G	0.3134	0.0345	0.0055	3.41E-10
rs3785074	TERF2	G	A	0.2628	0.0351	0.0056	4.50E-10
rs7194734	MPHOSPH6	T	C	0.7816	−0.0369	0.0060	6.72E-10
rs34978822	RTEL1	G	C	0.0148	−0.1397	0.0227	7.04E-10
rs34991172	CARMIL1	G	T	0.0684	−0.0608	0.0105	6.03E-09
rs228595	ATM	A	G	0.4169	−0.0285	0.0050	1.39E-08
rs2302588	DCAF4	C	G	0.1003	0.0476	0.0084	1.64E-08
rs13137667	MOB1B	C	T	0.9591	0.0765	0.0137	2.37E-08
rs55749605	SENP7	A	C	0.5790	−0.0373	0.0067	2.38E-08
rs62053580	RFWD3	G	A	0.1694	−0.0389	0.0071	3.96E-08
rs12909131	ATP8B4	T	C	0.2309	−0.0308	0.0058	1.15E-07
rs1744757	MROH8	T	C	0.8507	0.0359	0.0068	1.38E-07
rs2124616	TYMS	A	G	0.1400	−0.0374	0.0072	1.72E-07
rs2613954	RP11	T	C	0.8858	−0.0381	0.0078	1.10E-06
rs12065882	MAGI3	G	A	0.2084	0.0298	0.0062	1.36E-06
rs2386642	ASB13	A	G	0.6732	−0.0256	0.0053	1.44E-06
rs56810761	UNC80	T	C	0.2698	0.0275	0.0057	1.45E-06
rs62365174	TENT2	G	A	0.0882	−0.0544	0.0113	1.50E-06
rs112655343	ATF7IP	T	C	0.1017	0.0425	0.0090	2.22E-06
rs60160057	DCLK2	A	G	0.2115	−0.0287	0.0062	3.15E-06
rs117536281	CDCA4	G	A	0.0342	0.0850	0.0183	3.31E-06
rs59192843	BBOF1	G	T	0.0592	0.0655	0.0141	3.52E-06
rs57415150	CSMD1	A	G	0.0417	−0.0584	0.0126	3.68E-06
rs6038821	LINC01706	T	A	0.0383	0.0596	0.0129	3.98E-06
rs144204502	TK1	T	C	0.0142	−0.0896	0.0196	4.92E-06
rs6107615	PROKR2	C	T	0.4217	−0.0228	0.0050	5.30E-06
rs117037102	CEP295	T	C	0.0179	0.0979	0.0218	6.81E-06
rs7276273	KRTAP10-4	C	A	0.0074	−0.1502	0.0334	6.90E-06
rs11665818	IFNL2	A	G	0.1946	0.0278	0.0062	7.04E-06
rs3213718	CALM1	T	C	0.5828	0.0224	0.0050	7.22E-06
rs143276018	NMRK2	C	T	0.0182	−0.1015	0.0229	9.02E-06
rs7311314	SMUG1	A	G	0.3174	0.0240	0.0054	9.50E-06
rs35675808	CD247	G	C	0.0281	0.0736	0.0166	9.54E-06
rs117610974	UNC13C	G	C	0.0094	−0.1540	0.0350	1.05E-05

EA, indicates effect allele; NEA, non-effect allele; SE, standard error; SNP, single nucleotide polymorphism; EAF, effect allele frequency.

### 2.3 Skin Cancer and Skin Aging Genome-Wide Association Studies Selection

We searched the United Kingdom Biobank for aggregated GWAS data on common skin aging and cancers (NMSCs and melanoma) (Sudlow et al., 2015). Data on skin aging were obtained from a facial skin aging survey in 423,999 European participants; the skin cancer GWAS included 3,751 melanoma cases and 23,694 NMSCs, while 372,016 European participants were collected as controls. Analyses were adjusted for age, sex, and principal components when necessary. In addition, all SNPs in the MR analysis were derived from a GWAS of European ancestry to minimize potential bias due to population heterogeneity.

### 2.4 Single Nucleotide Polymorphisms Inclusion and Exclusion Criteria

To verify the validity of the IVs included in the MR analysis, we set the following screening criteria for eligible SNPs in the previously identified GWAS set. We selected SNPs that were significantly associated with our exposure (p ≤ 5E-8) and that had a certain probability of mutation (minor allele frequency, MAF ≥ 5%) with no reported locus coincidence. To estimate LD between SNPs, 1000 Genome Project samples were used (R^2^ < 0.01) (Siva, 2008). When there was an LD effect between SNPs, we selected the genetic variant with the lowest *p* value. We excluded all palindromic SNPs that could introduce ambiguity to the identity of the effector allele in the exposure GWAS. To limit bias from weak IVs, the F statistic should have been greater than 10. The formula for calculating F was as follows: R^2^ × (n−k−1)/[(1−R^2^) × k], where n is the sample size of the GWAS, k is the number of SNPs, and R^2^ is the proportion of telomere variability explained by each SNP. Specifically, the R formula calculates: 2 × beta^2^ × (1-EAF) × EAF, where EAF is the effect allele frequency, and beta is an estimate of the genetic effect of each SNP on TL. To satisfy the third core hypothesis, SNPs associated with skin aging, and skin cancer were excluded, as were pathways that did not include TL.

### 2.5 Method Selection

We estimated the risk relationship between TL and skin aging and cancer using the MR Egger, inverse variance weighted (IVW), weighted median, maximum likelihood, and weighted mode MR methods. Considering the potential pleiotropic genetic variation effects, to avoid bias, we focused on the MR-Egger regression results, the slope of which can estimate the directed pleiotropy magnitude. IVW is used to take a weighted average of random variable measurements. Each random variable is weighted using the inverse of its variance. This method minimizes the mean variance and is often used in meta-analyses to integrate independent measurement results. Maximum likelihood uses known sample results to infer the parameter values that are most likely (maximum probability) to lead to such an outcome, which outperforms naive regression methods and reduces bias in misspecification. In addition (Luque-Fernandez et al., 2018), we also performed weighted median and weighted mode analysis using IVs to accurately estimate causal effects for more than 50% of the weights. Results were presented as odds ratios (ORs) and 95% confidence intervals (CIs).

### 2.6 Sensitivity Analysis

A sensitivity analysis was performed using the heterogeneity, pleiotropy, and leave-one-out tests. First, the MR-Egger method was used to analyze pleiotropy and to verify whether a single locus affected multiple phenotypes. The pleiotropy refers to the phenomenon of a single locus affecting multiple phenotypes. Horizontal pleiotropy occurs when genetic variants are associated with multiple phenotypes along multiple pathways, which can invalidate results derived from MR analysis (Bowden et al., 2017). MR-Egger regression analysis can be used to evaluate the bias generated by horizontal pleiotropy, and its regression intercept can evaluate the size of pleiotropy. The closer the intercept is to 0, the smaller the possibility of gene pleiotropy. If *p* > 0.05, it is considered that the possibility of gene pleiotropy in the causal analysis is weak, and its effect can be ignored.Second, the SNPs were individually removed through a leave-one-out sensitivity test, and the drawn forest map was viewed after analysis. If a certain SNP is eliminated, the result changes greatly, indicating that this SNP is an outlier and needs to be eliminated. If the overall solid line do not change much after removing a certain SNP (all solid line are on the same side of 0), the results are reliable. Third, there may be heterogeneity in IVs from different platforms or populations affecting resultsthe. Combined MR-Egger, maximum likelihood, and IVW methods were used for heterogeneity analysis, and the Cochran Q statistic was used to standardize the heterogeneity analysis. We used a two-sided *p*-value, with statistical significance set at *p* < 0.05. Statistical analyses were performed using the “TwoSampleMR” package in R 3.4.2.

## 3 Result

**FIGURE 3:**
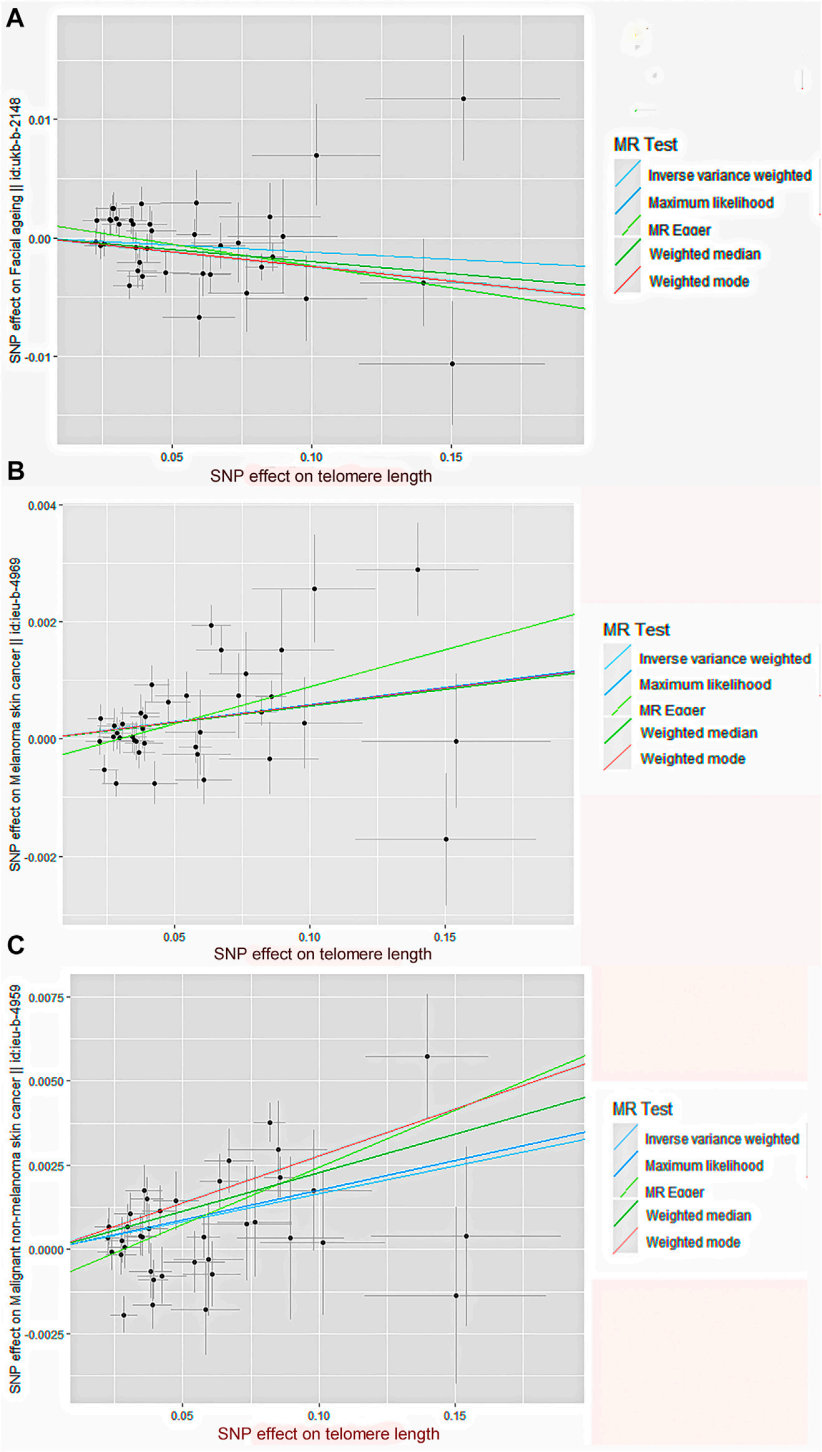
Scatter plot of genetic causality between TL and skin aging and cancer using different MR methods.**(A)** Skin aging; **(B)** Melanoma; **(C)** NMSCs. The slope of the line represents the causality of the different methods. The dark blue line represents Maximum likelihood, the light green line represents MR Egger, the dark green line represents Weighted median, the light blue line represents IVW, and the red line represents Weighted mode.

**FIGURE 4:**
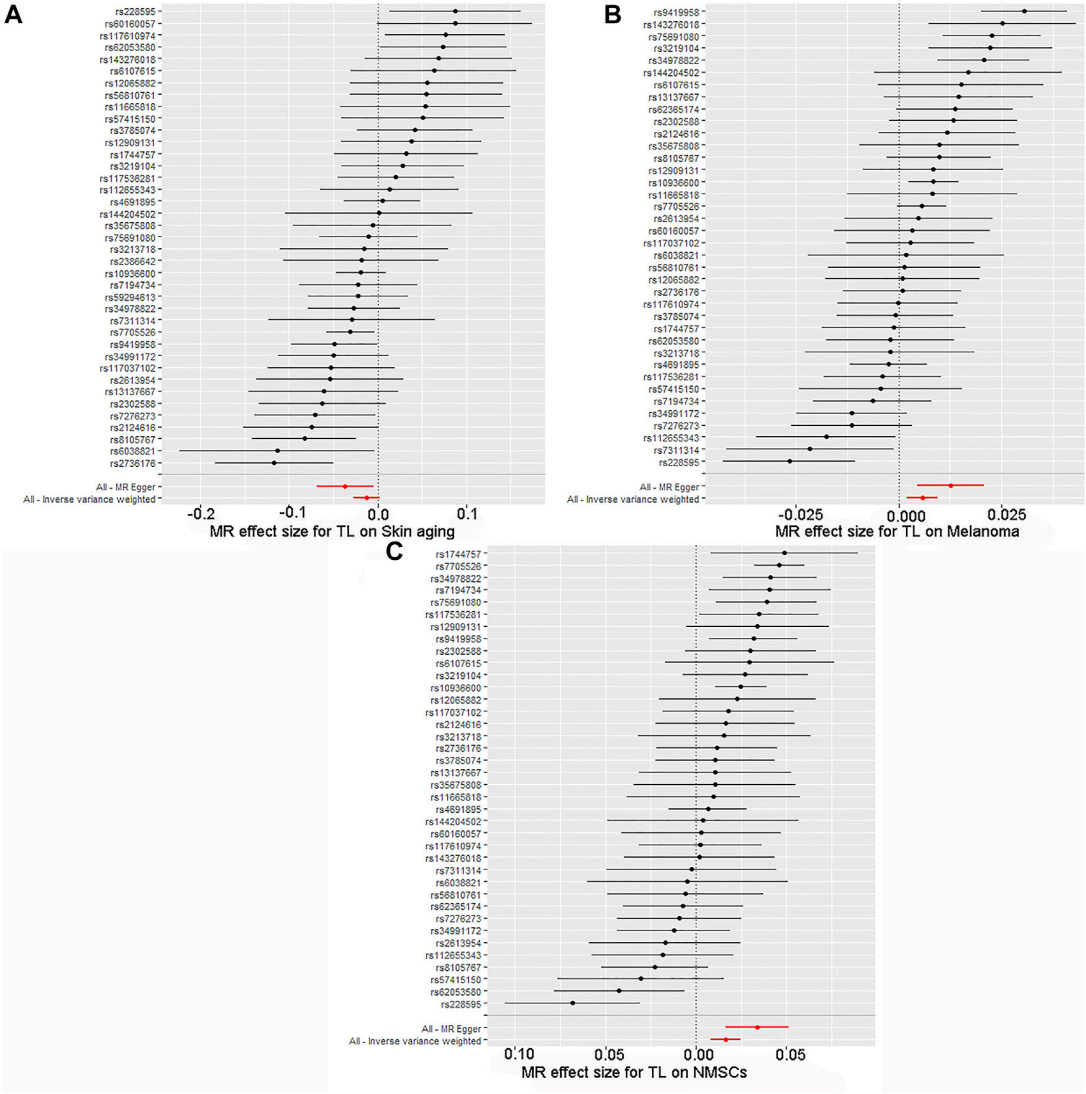
Forest map of skin cancer and aging risk based on TL genetic variants. **(A)** Skin aging; **(B)** Melanoma; **(C)** NMSCs.SNPs of TL were analyzed using IVW and MR-Egger. Black dots represent estimates of causal effects of TL on skin cancer and aging (beta coefficients). The black line represents the estimated 95% confidence interval.

By screening and selecting SNPs based on the above criteria, we identified 42 SNPs that met the TL criteria by means of multiple tests such as LD (Table 1). The analysis found that the causal relationship between TL and skin aging was consistent across the MR-Egger, weighted median, maximum likelihood, and weighted mode calculation methods.These scatterplots representing SNPs reflect the effects of TL on skin aging and skin cancer, as shown in Figure 3A. The results indicated that the risk of skin aging increased with TL shortening. The MR-Egger test showed that TL was significantly associated with skin aging (OR = 0.96, 95% CI: 0.9332–0.9956, *p* = 0.03). In addition, the weighted median, maximum likelihood, and weighted mode methods showed that TL shortening increased the risk of skin aging (Table 2). However, IVW did not reveal any causal link between TL and skin aging (OR = 0.9880, 95% CI: 0.9735–1.0027, *p* = 0.11). The IVW method produces consistent causal estimates by combining the Wald ratios of the causal effects of each SNP, but this may also introduce an invalid IV (Bowden et al., 2016). IVW methods are susceptible to being hampered by extreme propensity scores, leading to biased estimates and excessive variance (Li et al., 2019). Based on the above five analyses, we concluded that the causal relationship between TL and skin aging was significant. According to the forest map drawn by SNP (Figure 4), the analysis for skin cancer was adjusted to exclude four palindromic SNPs (rs55749605, rs59294613, rs2386642, and rs59192843). The risk relationship between TL and melanoma and NMSCs showed consistent results across the five methods. As shown by the MR-Egger method, TL was significantly associated with NMSCs (OR = 1.0344, 95% CI: 1.0168–1.0524, *p* = 4.60E-04) and melanoma (OR = 1.0127, 95% CI: 1.0046–1.0209, *p* = 6.36E-07). Three methods, including IVW, also demonstrated that the risk of both melanoma and NMSCs decreased with TL shortening. As shown in Figure 3, the five methods could intuitively determine the direction of agreement. Therefore, we concluded that TL was significantly associated with both melanoma and NMSCs.

**TABLE 2:** MR estimates for each method to assess the effect of TL on skin aging and skin cancer.

Outcome	MR Methods	Number of SNPs	OR (95%CI)	SE	*P*
Neuroblastoma	MR-Egger	39	0.9639 (0.9332∼0.9956)	0.0164	0.03
	Weighted median	39	0.9799 (0.9632∼0.9969)	0.0087	0.01
Simple mode	Inverse variance weighted	39	0.9880 (0.9735∼1.003)	0.0075	0.11
	Maximum likelihood	39	0.9878 (0.9777∼0.9982)	0.0053	0.02
	Weighted mode	39	0.9758 (0.9582∼0.9900)	0.0200	0.01
	MR-Egger	38	1.0344 (1.0168∼1.0524)	0.0088	4.60E-04
	Weighted median	38	1.0231 (1.0138∼1.0324)	0.0045	1.19E-06
	Inverse variance weighted	38	1.0166 (1.0084∼1.0249)	0.0041	6.33E-05
	Maximum likelihood	38	1.0178 (1.0126∼1.0231)	0.0027	1.09E-10
	Weighted mode	38	1.0282 (1.0120∼1.0447)	0.0079	1.79E-03
melanoma	MR-Egger	38	1.0127 (1.0046∼1.0209)	0.0041	6.36E-07
	Weighted median	38	1.0057 (1.0019∼1.0095)	0.0019	4.99E-03
	Inverse variance weighted	38	1.0057 (1.0020∼1.0095)	0.0019	2.68E-03
	Maximum likelihood	38	1.0059 (1.0036∼1.0082)	0.0011	6.36E-07
	Weighted mode	38	1.0058 (1.0017∼1.0103)	0.0023	1.96E-02

**FIGURE 5:**
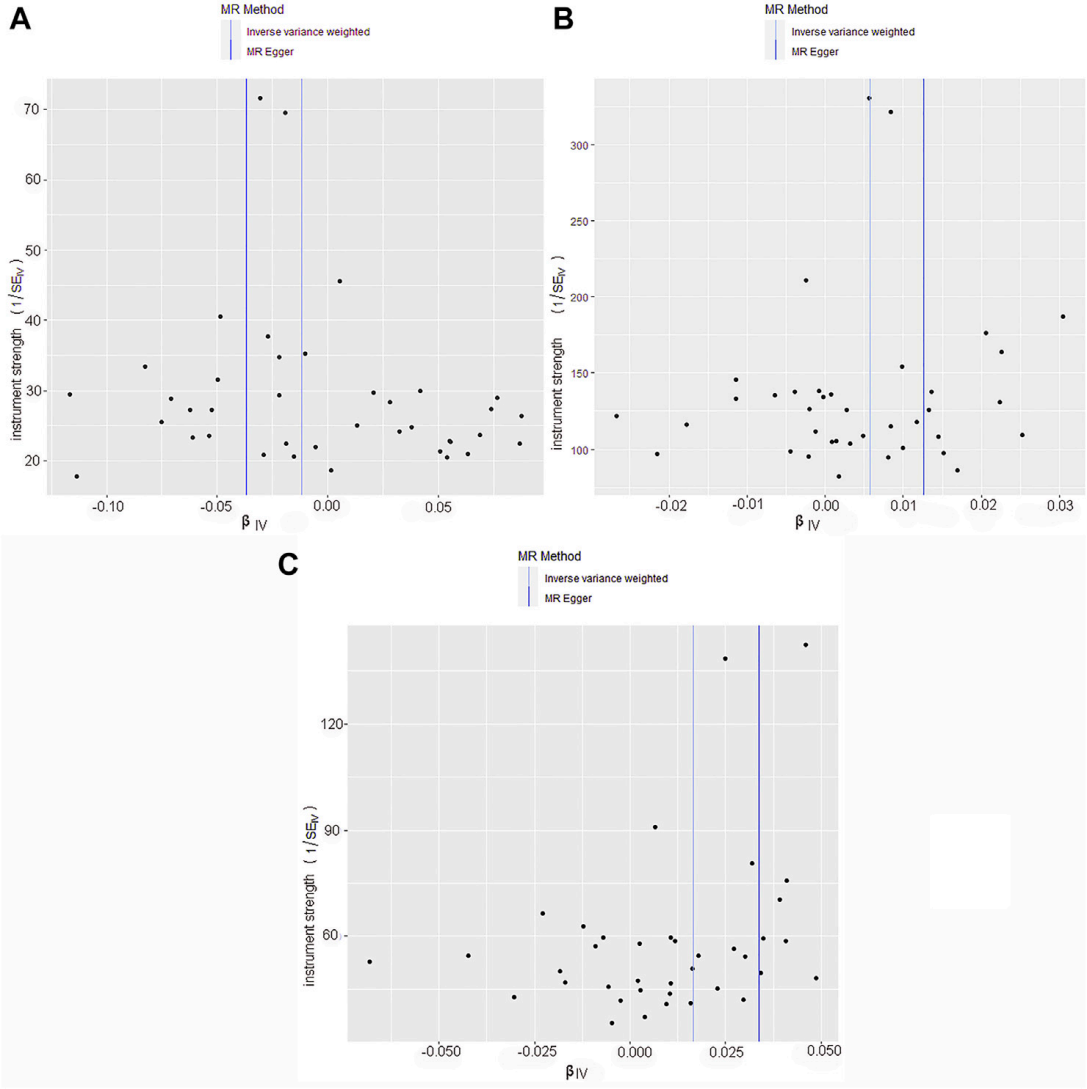
Funnel plot of skin cancer and aging risk based on TL genetic variants. **(A)** Skin aging; **(B)** Melanoma; **(C)** NMSCs.Causal effects were expressed as log odds ratios for skin aging and skin cancer per unit shortening of telomere length. Overall causal estimates (beta coefficients) of telomere length and skin aging and skin cancer estimated by the IVW (light blue line) and MR-Egger (dark blue line) methods are shown.

**FIGURE 6:**
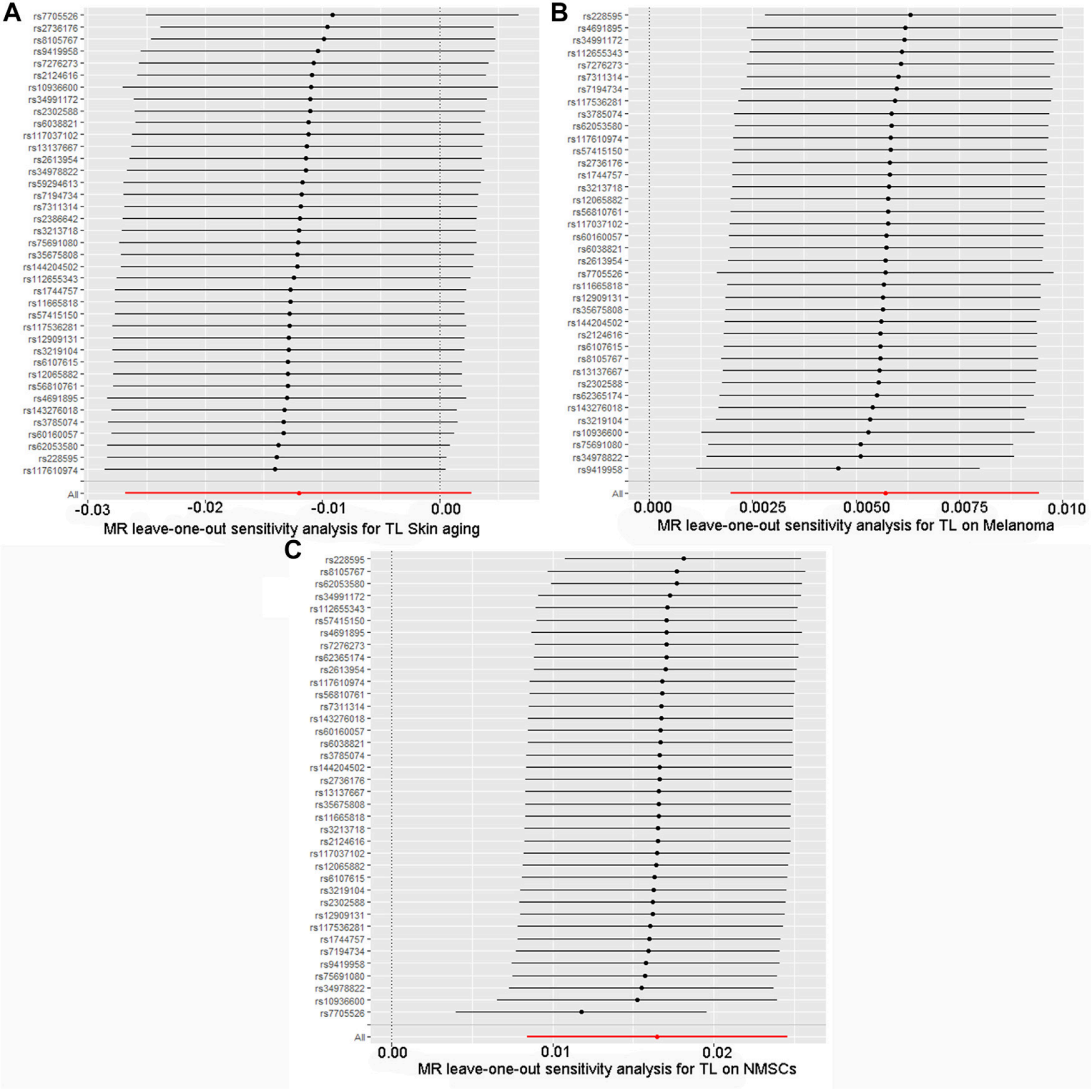
Forest plot for leave-one-out sensitivity analysis. **(A)** Skin aging; **(B)** Melanoma; **(C)** NMSCs.Each horizontal solid line reflects the result estimated by a single SNP using the Wald ratio method.The solid line is completely to the left of 0, indicating that the estimated result from this SNP is that TL shortening can increase skin aging. The solid line is completely to the right of 0, indicating that the estimated result from this SNP is that TL shortening can reduce the risk of skin cancer. All SNPs are on the side of 0, representing the stabilityof the results.

The quality controls for this study included pleiotropy, heterogeneity, and sensitivity tests. MR-Egger regression was used to test the pleiotropic effects of TL on skin aging, NMSCs, and melanoma. The results of each group showed that the effect of TL on skin aging and skin cancer had no significant horizontal pleiotropic bias (skin aging, *p* = 0.1; melanoma, *p* = 0.72; NMSCs, *p* = 0.72) (Table 3). The funnel plot according to IVW and MR-Egger also suggests that there was no horizontal pleiotropy (Figure 5). We utilized three algorithms (MR-Egger, maximum likelihood, and IVW) to analyze whether there was statistical heterogeneity among the IV estimates. We found that there was substantial heterogeneity among these IVs across the three outcomes (i.e., for skin aging, MR-Egger Q = 76.72, *p* = 1.36E-04; for melanoma, MR-Egger Q = 97.10, *p* = 1.62E-07; for NMSCs, MR-Egger Q = 538.50, *p* = 1.90E-05). We analyzed the sensitivity by performing the leave-one-out sensitivity test and found that regardless of which SNP was removed, it would not fundamentally impact the results (all lines were on the same side of 0) (Figure 6), This indicated that the MR results were robust. The solid line is completely to the left of 0, indicating that the estimated result from this SNP is that TL shortening can increase skin aging. The solid line is completely to the right of 0, indicating that the estimated result from this SNP is that TL shortening can reduce the risk of skin cancer. All SNPs are on the side of 0, representing the stabilityof the results.In Figure 6A, the solid line is completely to the left of 0, indicating that the estimated result for this SNP is that TL shortening increases skin aging. In Figure 6B,C, the solid line is completely to the right of 0, indicating that the estimated result for this SNP is that TL shortening reduces the risk of skin cance.

**TABLE 3:** Heterogeneity and pleiotropy analysis by MR Egger, IVW, Maximum likelihood.

Outcome	MR Methods	Cochran Q statistic	Heterogeneity *p*-value	Pleiotropy *p*-value
skin aging	MR-Egger	76.72	1.36E-04	0.10
	Inverse variance weighted	82.55	3.86E-05	
	Maximum likelihood	82.30	4.15E-05	
NMSCs	MR-Egger	82.02	1.90E-05	0.10
	Inverse variance weighted	93.12	9.78E-07	
	Maximum likelihood	90.62	2.15E-06	
melanoma	MR-Egger	97.10	1.62E-07	0.72
	Inverse variance weighted	106.60	1.15E-08	
	Maximum likelihood	105.44	1.71E-08	

## 4 Discussion

Telomere shortening is observed in most human cancers, but it is worth noting that this phenomenon is controversial.New research suggests that longer-than-expected telomeres (made up of repetitive DNA sequences) are associated with an increased risk of several cancers, including melanoma (De Vitis et al., 2018). A novel point of this study is the paradoxical issue of factor analysis from genetics.Exploring epigenetic drivers of skin cancer and skin aging by means of TL-related SNPs.There are 42 SNPs that met the inclusion criteria for the core assumption of MR. Utilizing these IVs not only enables inferring a causal relationship between outcomes and exposure, but also effectively avoids confounding bias in traditional epidemiological studies (Tin and Köttgen, 2021). For example, the two SNPs (rs7705526, rs4691895) that we screened for maintaining TL are located near the TERT gene (Table 1). TERT is a cancer-related gene, and mutations in the noncoding region of the TERT gene are considered to be the cause of most melanomas (Toussi et al., 2020). The second innovation of this study, the MR study used an epidemiological approach to explore the association between skin aging and skin cancer. This study uses TL genetic variants as probes, reduces confounding factors and reverse causality and may be more convincing than classical observational experiments (Davey Smith and Hemani, 2014).

Telomeres are nuclear protein complexes at chromosome ends that maintain chromosomal stability, upon which normal cell division, differentiation, and regeneration depend (Turner et al., 2019). Normal telomeres and telomerase can regulate skin cell physiological function and abnormal proliferation; therefore, TLs play an essential role in skin aging and cancer development (Ventura et al., 2019). We used a two-sample MR to assess the association between TL and skin aging and cancer. We found an increased likelihood of skin aging with TL shortening. In contrast, the risk of NMSCs and melanoma was significantly reduced with TL shortening. Contrary to the general belief that cell senescence can trigger cancer, TL shortening as a risk factor for skin aging did not increase the risk of skin cancer. This is consistent with recent clinical reports that long telomeres are associated with increased mortality in more than 2000 melanoma patients from hospital clinics and the general population (Ismail et al., 2021).

Telomeres are closely linked to cellular aging, especially in dermal cells. Telomere shortening in skin fibroblasts may lead to epidermal aging and barrier function defects (Quan et al., 2015). Long-term exposure to ultraviolet (UVA) radiation has long been recognized as the most important factor in skin aging. Studies have found that in fibroblasts exposed to UVA at doses of 1000 or 10,000 mJ/cm^2^, TL was significantly shorter than in unirradiated controls, negatively correlating with the UVA dose (Ma et al., 2012). In addition, reactive oxygen species (ROS) are considered another cause of skin aging (Kammeyer and Luiten, 2015). Studies have shown that ROS cause cellular senescence owing to accelerated telomere shortening (Anderson et al., 2014). In the present study, all four MR methods showed a significant relationship between TL shortening and skin aging. Each standard deviation of TL shortening was genetically predicted to increase the risk of skin aging by 4.61%. The sensitivity analysis verified the reliability of all SNP results using the leave-one-out method. Therefore, maintaining TL can protect chromosome stability and prevent skin aging caused by DNA breakage damage.

TL is a key factor in cell proliferative potential, and much evidence supports the vital role of altered TL in cancer pathogenesis (Xu et al., 2013). However, studies analyzing the association between TL and cancer risk have yielded conflicting conclusions (Aviv et al., 2017). Studies have found that most solid cancers originating from proliferating tissues display short telomere characteristics, and most cancer incidence increases with age. In contrast, in the general population, individuals with constitutively long telomeres also have an increased risk of some serious cancers (Savage et al., 2013). From Table 1, the genes of SNPs that maintain TL are all related to cancer. For example, the protection of telomere 1 (POT1) protein is an important subunit of the Shelterin telomere-binding complex, which can promote the development of various cancers by leading to immortalization (Wu et al., 2020). Therefore, combined with the multiple related genes we screened (including STN1, STMN3, PRRC2A), we speculate that the telomere shortening inhibitory pathway in tumors has been determined at birth.when telomeres are too long, the telomere reserve will not be depleted in time, which will provide additional divisions for cancer cells, especially for familial-prone tumors (melanoma glioma, non-Hodgkin lymphoma, etc.) (Nieters et al., 2012; Feng et al., 2018; Ali et al., 2021).

### 4.1 Limitations

Because of the small study population, the effect of TL on skin aging and cancer was not found to be very significant. To bring our results closer to reality, we used five algorithms with different characteristics, such as the MR-Egger method, as a reference. This method assessed whether genetic variation had pleiotropic effects on the results that differed from zero on average (directional pleiotropy) and provided consistent causal effect estimates under a weaker assumption–the InSIDE (instrumental strength independent of direct effects) assumption (Burgess and Thompson, 2017). Furthermore, we selected the largest and most reliable GWAS available to explore the causal relationship between TL and skin aging and cancer. In our MR research framework, the interference of confounders and reverse causality were minimized. In addition, the original United Kingdom Biobank study lacked a breakdown of the population (including gender and age) and was unable to conduct further subgroup analyses. Clinically, the complex physiological mechanisms of TL and skin aging and cancer go well beyond these simple models. Further studies are needed to identify the underlying mechanisms that provide insights into skin aging and cancer and to facilitate prevention.

## 5 Conclusion

This study supported the causal relationship that TL shortening may promote the development of skin aging and reduce the risk of cutaneous melanoma and NMSCs. The results provide a reference for future research on the relationship between skin aging and cancer in clinical practice. Also, our study provides evidence for skin aging and cancer (melanoma and NMSCs) treatment and diagnosis.

## Data Availability

The original contributions presented in the study are included in the article/supplementary material, further inquiries can be directed to the corresponding authors.

## References

[B1] Ali M. W. Patro C. P. K. Zhu J. J. Dampier C. H. Plummer S. J. Kuscu C. (2021). A Functional Variant on 20q13.33 Related to Glioma Risk Alters Enhancer Activity and Modulates Expression of Multiple Genes. Hum. Mutat. 42 (1), 77–88. 10.1002/humu.24134 33169458 PMC7839675

[B2] Anderson A. Bowman A. Boulton S. J. Manning P. Birch-Machin M. A. (2014). A Role for Human Mitochondrial Complex II in the Production of Reactive Oxygen Species in Human Skin. Redox Biol. 2, 1016–1022. 10.1016/j.redox.2014.08.005 25460738 PMC4215388

[B3] Arsenis N. C. You T. Ogawa E. F. Tinsley G. M. Zuo L. (2017). Physical Activity and Telomere Length: Impact of Aging and Potential Mechanisms of Action. Oncotarget 8 (27), 45008–45019. 10.18632/oncotarget.16726 28410238 PMC5546536

[B4] Aviv A. Anderson J. J. Shay J. W. (2017). Mutations, Cancer and the Telomere Length Paradox. Trends Cancer 3 (4), 253–258. 10.1016/j.trecan.2017.02.005 28718437 PMC5903276

[B5] Blasco M. A. (2005). Telomeres and Human Disease: Ageing, Cancer and beyond. Nat. Rev. Genet. 6 (8), 611–622. 10.1038/nrg1656 16136653

[B6] Bowden J. Holmes M. V. (2019). Meta‐analysis and Mendelian Randomization: A Review. Res. Syn. Meth 10 (4), 486–496. 10.1002/jrsm.1346 30861319 PMC6973275

[B7] Bowden J. Davey Smith G. Haycock P. C. Burgess S. (2016). Consistent Estimation in Mendelian Randomization with Some Invalid Instruments Using a Weighted Median Estimator. Genet. Epidemiol. 40 (4), 304–314. 10.1002/gepi.21965 27061298 PMC4849733

[B8] Bowden J. Del Greco M F. Minelli C. Davey Smith G. Sheehan N. Thompson J. (2017). A Framework for the Investigation of Pleiotropy in Two-Sample Summary Data Mendelian Randomization. Stat. Med. 36 (11), 1783–1802. 10.1002/sim.7221 28114746 PMC5434863

[B9] Buckingham E. M. Klingelhutz A. J. (2011). The Role of Telomeres in the Ageing of Human Skin. Exp. Dermatol. 20 (4), 297–302. 10.1111/j.1600-0625.2010.01242.x 21371125 PMC3690281

[B10] Burgess S. Thompson S. G. (2017). Interpreting Findings from Mendelian Randomization Using the MR-Egger Method. Eur. J. Epidemiol. 32 (5), 377–389. 10.1007/s10654-017-0255-x 28527048 PMC5506233

[B11] Cheng Q. Yang Y. Shi X. Yeung K.-F. Yang C. Peng H. (2020). MR-LDP: a Two-Sample Mendelian Randomization for GWAS Summary Statistics Accounting for Linkage Disequilibrium and Horizontal Pleiotropy. Nar. Genom Bioinform. 2 (2), lqaa028. 10.1093/nargab/lqaa028 33575584 PMC7671398

[B12] Davey Smith G. Hemani G. (2014). Mendelian Randomization: Genetic Anchors for Causal Inference in Epidemiological Studies. Hum. Mol. Genet. 23 (R1), R89–R98. 10.1093/hmg/ddu328 25064373 PMC4170722

[B13] De Vitis M. Berardinelli F. Sgura A. (2018). Telomere Length Maintenance in Cancer: At the Crossroad between Telomerase and Alternative Lengthening of Telomeres (ALT). Int. J. Mol. Sci. 19 (2), 606. 10.3390/ijms19020606 29463031 PMC5855828

[B14] Feng X. Hsu S.-J. Bhattacharjee A. Wang Y. Diao J. Price C. M. (2018). CTC1-STN1 Terminates Telomerase while STN1-TEN1 Enables C-Strand Synthesis during Telomere Replication in Colon Cancer Cells. Nat. Commun. 9 (1), 2827. 10.1038/s41467-018-05154-z 30026550 PMC6053418

[B15] Ismail H. Helby J. Hölmich L. R. Chakera A. H. Bastholt L. Klyver H. (2021). Genetic Predisposition to Long Telomeres Is Associated with Increased Mortality after Melanoma: A Study of 2101 Melanoma Patients from Hospital Clinics and the General Population. Pigment. Cell Melanoma Res. 34 (5), 946–954. 10.1111/pcmr.12971 33749133

[B16] Kammeyer A. Luiten R. M. (2015). Oxidation Events and Skin Aging. Ageing Res. Rev. 21, 16–29. 10.1016/j.arr.2015.01.001 25653189

[B17] Li F. Thomas L. E. Li F. (2019). Addressing Extreme Propensity Scores via the Overlap Weights. Am. J. Epidemiol. 188 (1), 250–257. 10.1093/aje/kwy201 30189042

[B18] Li C. Stoma S. Lotta L. A. Warner S. Albrecht E. Allione A. (2020). Genome-wide Association Analysis in Humans Links Nucleotide Metabolism to Leukocyte Telomere Length. Am. J. Hum. Genet. 106 (3), 389–404. 10.1016/j.ajhg.2020.02.006 32109421 PMC7058826

[B19] Linares M. A. Zakaria A. Nizran P. (2015). Skin Cancer. Prim. Care Clin. Office Pract. 42 (4), 645–659. 10.1016/j.pop.2015.07.006 26612377

[B20] Lowry W. (2020). Its Written All over Your Face: The Molecular and Physiological Consequences of Aging Skin. Mech. Ageing Dev. 190, 111315. 10.1016/j.mad.2020.111315 32681843 PMC8911920

[B21] Luque-Fernandez M. A. Schomaker M. Rachet B. Schnitzer M. E. (2018). Targeted Maximum Likelihood Estimation for a Binary Treatment: A Tutorial. Stat. Med. 37 (16), 2530–2546. 10.1002/sim.7628 29687470 PMC6032875

[B22] Ma H.-M. Liu W. Zhang P. Yuan X.-Y. (2012). Human Skin Fibroblast Telomeres Are Shortened after Ultraviolet Irradiation. J. Int. Med. Res. 40 (5), 1871–1877. 10.1177/030006051204000526 23206469

[B23] Morrison J. Knoblauch N. Marcus J. H. Stephens M. He X. (2020). Mendelian Randomization Accounting for Correlated and Uncorrelated Pleiotropic Effects Using Genome-wide Summary Statistics. Nat. Genet. 52 (7), 740–747. 10.1038/s41588-020-0631-4 32451458 PMC7343608

[B24] Nieters A. Conde L. Slager S. L. Brooks-Wilson A. Morton L. Skibola D. R. (2012). PRRC2A and BCL2L11 Gene Variants Influence Risk of Non-Hodgkin Lymphoma: Results from the InterLymph Consortium. Blood 120 (23), 4645–4648. 10.1182/blood-2012-05-427989 23047821 PMC3512239

[B25] Quan C. Cho M. K. Perry D. Quan T. (2015). Age-associated Reduction of Cell Spreading Induces Mitochondrial DNA Common Deletion by Oxidative Stress in Human Skin Dermal Fibroblasts: Implication for Human Skin Connective Tissue Aging. J. Biomed. Sci. 22 (1), 62. 10.1186/s12929-015-0167-6 26215577 PMC4517525

[B26] Savage S. A. Gadalla S. M. Chanock S. J. (2013). The Long and Short of Telomeres and Cancer Association Studies. JNCI J. Natl. Cancer Inst. 105 (7), 448–449. 10.1093/jnci/djt041 23468461 PMC3614507

[B27] Shay J. W. (2018). Telomeres and Aging. Curr. Opin. Cell Biol. 52, 1–7. 10.1016/j.ceb.2017.12.001 29253739

[B28] Siva N. (2008). 1000 Genomes Project. Nat. Biotechnol. 26 (3), 256. 10.1038/nbt0308-256b 18327223

[B29] Sudlow C. Gallacher J. Allen N. Beral V. Burton P. Danesh J. (2015). UK Biobank: an Open Access Resource for Identifying the Causes of a Wide Range of Complex Diseases of Middle and Old Age. PLoS Med. 12 (3), e1001779. 10.1371/journal.pmed.1001779 25826379 PMC4380465

[B30] Tin A. Köttgen A. (2021). Mendelian Randomization Analysis as a Tool to Gain Insights into Causes of Diseases: A Primer. J. Am. Soc. Nephrol. 32 (10), 2400–2407. 10.1681/ASN.2020121760 34135084 PMC8722812

[B31] Toussi A. Mans N. Welborn J. Kiuru M. (2020). Germline Mutations Predisposing to Melanoma. J. Cutan. Pathol. 47 (7), 606–616. 10.1111/cup.13689 32249949 PMC8232041

[B32] Turner K. Vasu V. Griffin D. (2019). Telomere Biology and Human Phenotype. Cells 8 (1), 73. 10.3390/cells8010073 30669451 PMC6356320

[B33] Ventura A. Pellegrini C. Cardelli L. Rocco T. Ciciarelli V. Peris K. (2019). Telomeres and Telomerase in Cutaneous Squamous Cell Carcinoma. Int. J. Mol. Sci. 20 (6), 1333. 10.3390/ijms20061333 30884806 PMC6470499

[B34] Wu Y. Poulos R. C. Reddel R. R. (2020). Role of POT1 in Human Cancer. Cancers 12 (10), 2739. 10.3390/cancers12102739 32987645 PMC7598640

[B35] Xu L. Li S. Stohr B. A. (2013). The Role of Telomere Biology in Cancer. Annu. Rev. Pathol. Mech. Dis. 8, 49–78. 10.1146/annurev-pathol-020712-164030 22934675

