# Correction: Association between telomere length and skin cancer and aging: a mendelian randomization analysis

**DOI:** 10.3389/fgene.2026.1821032

**Published:** 2026-03-13

**Authors:** Nannan Song, Yankun Cui, Wang Xi

**Affiliations:** Jiangxi University of Chinese Medicine, Nanchang, China

**Keywords:** telomere length, skin cancer, skin aging, Mendelian randomization, age

The funder Science and Technology Project (Youth Project) of Jiangxi Provincial Department of Education, No. GJJ201260 to author WX was erroneously omitted.

The original article has been updated.

